# A *WOX5/7–SCR* Reciprocal Feedback Loop in Middle Cell Layer Drives Callus Proliferation

**DOI:** 10.3390/plants15020210

**Published:** 2026-01-09

**Authors:** Aoyun Pang, Yajie Li, Chongzhen He, Caifeng Liu, Hongpei Jin, Limin Pi, Yi Yang

**Affiliations:** 1 State Key Laboratory of Hybrid Rice, Institute for Advanced Studies, Wuhan University, Wuhan 430072, China; 2 Xiaogan Academy of Agricultural Sciences, Xiaogan 432100, China; 3 Guangxi Salt-Alkali Tolerant Food Crop Seed Engineering Research Center, College of Agricultural Engineering, Guangxi Vocational University of Agriculture, Nanning 530007, China

**Keywords:** *WUSCHEL-RELATED HOMEOBOX 5/7*, *SCARECROW*, cell cycle, callus, pluripotency, middle cell layer

## Abstract

In plant tissue culture, the middle cell layer of the callus is crucial for establishing pluripotency and serves as the foundation for subsequent organ regeneration. Although several root apical stem cell regulators have been implicated in maintaining callus pluripotency, how they functionally coordinate to control the formation and proliferation of the middle callus layer remains unclear. Here, we identify a reciprocal transcriptional activation between the root stem cell regulators *WOX5/7* and *SCR* in the middle callus layer of *Arabidopsis*. We further show that WOX5/7 and SCR form protein complexes that mutually enhance their transcriptional activities. Transcriptomic analysis reveals that *WOX5/7* and *SCR* co-regulate a subset of cell cycle-related genes, explaining the reduced mitotic activity observed in the callus of *wox5 wox7* double mutants and *scr* mutants. Together, these findings support a model in which *WOX5/7* and *SCR* establish a reciprocal positive feedback loop in the middle cell layer that drives the robust callus proliferation by promoting cell cycle progression.

## 1. Introduction

In classic plant in vitro culture systems, callus tissue is induced from explants—such as roots, hypocotyls, and cotyledons—by culturing on callus induction medium (CIM) with an appropriate ratio of auxins and cytokinins [1]. Originally regarded as a disorganized cell mass, the callus is now recognized as a heterogeneous tissue. Its development follows a pattern similar to that of lateral root primordia, originating from pericycle or pericycle-like cells adjacent to xylem poles [2,3]. However, unlike the stereotypic development of lateral roots, callus ontogeny exhibits greater complexity and randomness [4].

Recent advances in single-cell transcriptomics have revealed that the early callus can be systematically partitioned into three distinct layers perpendicular to the explant’s vascular axis: the outer, middle, and inner layers [5,6]. The outer layer expresses epidermal-related genes, while the inner layer is marked by vascular and provascular gene expression. The middle layer is characterized by the enrichment of quiescent center (QC)-like identity genes, and lineage tracing has confirmed that regenerated organs originate specifically from this middle domain [5,7]. Thus, the establishment and proliferative activity of the middle layer are critical for the success of de novo organogenesis.

Several transcription factors play important roles in maintaining the identity of the QC in the root and the middle cell layer in the callus. Among them, the homeodomain transcription factor *WUSCHEL-RELATED HOMEOBOX5* (*WOX5*), which is specifically expressed in the QC, is an essential regulator of stem cell activity [8,9]. *WOX5* inhibits QC division and prevents stem cell differentiation by suppressing *D-type CYCLIN* (*CYCD*) genes and *CYCLING DOF FACTOR4* (*CDF4*), respectively [8,10]. The GRAS transcription factor SCARECROW (SCR), which is involved in positioning the root stem cell niche [11,12], activates *WOX5* expression by recruiting the epigenetic co-regulator SEUSS (SEU) to its promoter [13]. Furthermore, SCR can form higher-order complexes with plant-specific teosinte-branched cycloidea PCNA (TCP) transcription factors and AP2-family transcription factors PLETHORAs (PLTs) to guide *WOX5* expression, thereby establishing the root stem cell niche [14].

During callus development, the GRAS-family protein SHORT-ROOT (SHR) is expressed in the inner layer and moves into the middle layer, where it interacts with SCR. This complex activates the expression of *WOX5* and its closest homolog *WOX7* (collectively *WOX5/7*) [5,15]. *WOX5/7* in turn promotes auxin synthesis and modulates cytokinin responses, thereby sustaining pluripotency in the middle layer [5,15]. Mutations in any of these genes cause severe regeneration defects [16]. Although recent work has greatly advanced our understanding of pluripotency establishment in callus, how these key regulators coordinate to control callus growth remains poorly understood.

Previous studies have shown that *WOX5/7* maintain callus development by regulating cell cycle genes, underscoring their crucial role in sustaining the division activity of the pluripotent middle cell layer. Here, using a combination of molecular, biochemical, and microscopy approaches, we further investigated the molecular mechanism by which *WOX5/7* form a reciprocal regulatory loop with *SCR* to control cell proliferation during callus formation.

## 2. Materials and Methods

### 2.1. Plant Materials

The *Arabidopsis thaliana* ecotype Col-0 was used as the wild-type control in this study. The following previously described mutants and transgenic lines were utilized: *SCRpro:SCR-eGFP* [17], *WOX5pro:NLS-3×eGFP* [15], *wox5-1 wox7-1 double mutant* [18], and *scr-6* [16]. See Table S2 for a list of plant materials used in this study.

### 2.2. Callus Induction

*Arabidopsis* seeds were surface-sterilized with 10% sodium hypochlorite and sown on half-strength Murashige and Skoog (1/2 MS) basal medium without sucrose. The plates were incubated at 22 °C in darkness. Five days after germination, 1 cm hypocotyl explants were excised from the seedlings and transferred to callus induction medium (CIM), which consisted of MS basal medium supplemented with vitamins, 2% (*w*/*v*) sucrose, 11 μM 2,4-dichlorophenoxyacetic acid (2,4-D), and 0.2 μM kinetin. The pH of the medium was adjusted to 5.7 before solidification with 0.8% (*w*/*v*) agar. Cultures were maintained at 22 °C under continuous light, with a light intensity of 100–150 μmol·m^−2^·s^−1^ provided by a combination of cool-white fluorescent lamps and incandescent bulbs to deliver a broad spectrum. Relative humidity was controlled at 60–70%.

### 2.3. Y2H Assay

For the yeast two-hybrid (Y2H) assay, the coding sequences (CDS) of *WOX5* and *WOX7* were cloned into the pGBKT7 bait vector, while the CDS of *SCR* and *SHR* were cloned into the pGADT7 prey vector. The assay was conducted following a previously established method [19]. All experiments were independently replicated at least three times with consistent results. All primer sequences used in this assay are provided in Table S1.

### 2.4. BiFC Assay

Bimolecular Fluorescence Complementation (BiFC) was employed to validate protein–protein interactions in vivo. The *SCR* coding sequence was fused to the C-terminal fragment of YFP in the pSPYCE vector, and the coding sequences of *WOX5* and *WOX7* were separately fused to the N-terminal fragment of YFP in the pSPYNE vector. See Table S2 for a list of constructs used in this study. These constructs were transiently co-expressed in *Arabidopsis* leaf protoplasts, and the assay was conducted following an established protocol [20]. Each interaction pair was tested in at least three independent experiments.

### 2.5. EdU Staining and Calcofluor White Staining

The hypocotyl explants cultured on CIM for 7 days were transferred to CIM supplemented with 2.5 mM EdU (UElandy, C6044L), and grown for 4 h. Subsequent EdU detection and cell wall staining were performed following an established protocol [21].

### 2.6. Microscopy

Confocal microscopy was performed using a Leica SP8 confocal microscope (Leica Microsystems, Wetzlar, Germany). The fluorescent proteins were detected with the following spectral settings: GFP was excited at 488 nm with emission collected between 500 and 540 nm; PI was excited at 554 nm with emission collected between 570 and 620 nm.

To analyze the area of callus, hypocotyl explants grown vertically on CIM were harvested and subjected to clearing as previously described [5]. The callus treated on CIM for 11 days was imaged and quantified on a Leica DM2500 differential interferometric contrast microscope (DIC) using LAS V4.12 software.

### 2.7. Dual-Luciferase Reporter Assay

Firefly luciferase reporter vectors were constructed by cloning the 3 kb promoters of *SCR*, *WOX5*, and *WOX7* into the pGreenII 0800-LUC vector. The full-length coding sequences of *GUS*, *WOX5*, *WOX7*, and *SCR* were cloned into the pGreenII 62-SK vector to generate effectors. See Table S2 for a list of constructs used in this study. For transient expression assays, the reporter and effector plasmids were co-transformed with the p19 silencing suppressor into young leaves of *Nicotiana benthamiana* via *Agrobacterium tumefaciens* infiltration. The infiltrated leaves were harvested 48 h post-infiltration. Firefly luciferase (LUC) and Renilla luciferase (REN) activities were measured using a Dual-Luciferase Reporter Assay Kit (Vazyme, DL101-01). The relative LUC activity was represented by the ratio of LUC to REN luminescence.

### 2.8. Reverse Transcription Quantitative Real-Time PCR

Total RNA was isolated from 5-day-old calli cultured on callus induction medium (CIM) using the FastPure Universal Plant Total RNA Isolation Kit (Vazyme, RC411). First-strand cDNA was synthesized from the extracted RNA using the TransScript Uni All-in-One First-Strand cDNA Synthesis SuperMix for qPCR (TransGen, AU341). Quantitative real-time PCR was then performed with the PerfectStart Green qPCR SuperMix (TransGen, AQ601) on a CFX Real-Time PCR Detection System (Bio-Rad, Hercules, CA, USA). The RT-qPCR thermal cycling protocol was as follows: initial denaturation at 94 °C for 30 s; followed by 40 cycles of denaturation at 94 °C for 5 s, annealing/extension at 60 °C for 30 s; and a final melting curve analysis step (94 °C for 15 s, 60 °C for 1 min, then continuous heating to 94 °C with fluorescence measurement) to verify amplicon specificity. The *ACTIN2* gene (*AT3G18780*) was used as an internal control for normalization. Gene expression levels were calculated using the ΔCT method in Microsoft Excel. All primer sequences used in this assay are provided in Table S1.

### 2.9. RNA-Seq Analysis

Hypocotyl explants were collected from 5-day-old calli grown on CIM from the *Arabidopsis* Col-0 wild type, *wox5-1 wox7-1* double mutant, and *scr-6* mutant. For each genotype, three biological replicates were prepared, with each replicate comprising a pool of 40 explants. Total RNA was extracted using the RNeasy Plant Mini Kit (QIAGEN (Hilden, Germany), #74903), and RNA integrity was assessed using an Agilent 4200 Bioanalyzer (Santa Clara, CA, USA)_. Library construction and sequencing were performed by Berry Genomics (Beijing, China).

Raw sequencing reads were processed using fastp (v0.23.4) for adapter removal and quality filtering with the parameters: -q 20-u 30-n 5-w 8. Clean reads were then aligned to the *Arabidopsis thaliana* TAIR10 reference genome using STAR (v2.7.3a) in two-pass mode (--twopassMode Basic).

Gene expression quantification was performed using RSEM (v1.3.1) based on the aligned BAM files to obtain raw read counts and fpkm values. Differential expression analysis was conducted in R (v4.2.0) using the DESeq2 package (v1.38.3). Genes with an *p*-adj ≤ 0.05 and |log_2_(fold change)| ≥ 1 were considered significantly differentially expressed. Principal component analysis (PCA) and hierarchical clustering were performed on variance-stabilizing transformed (VST) expression values to evaluate sample reproducibility and transcriptome similarity across genotypes.

Gene ontology (GO) enrichment analysis of differentially expressed genes was performed using Metascape (https://metascape.org), a web-based tool for gene annotation and analysis resource [22]. Enrichment results were visualized using dot plots to highlight biological processes associated with differentially expressed genes.

### 2.10. Statistical Analysis

The callus area was measured using ImageJ 1.54 software, and statistical analysis was performed with GraphPad Prism 9.0. Statistical significance, as indicated in the figure legends, was determined using a Student’s *t*-test. Significance levels were defined as * *p* < 0.05, ** *p* < 0.01, and *** *p* < 0.001.

## 3. Results

### 3.1. Mutual Transcriptional Regulation Between WOX5/7 and SCR

**Figure 1:**
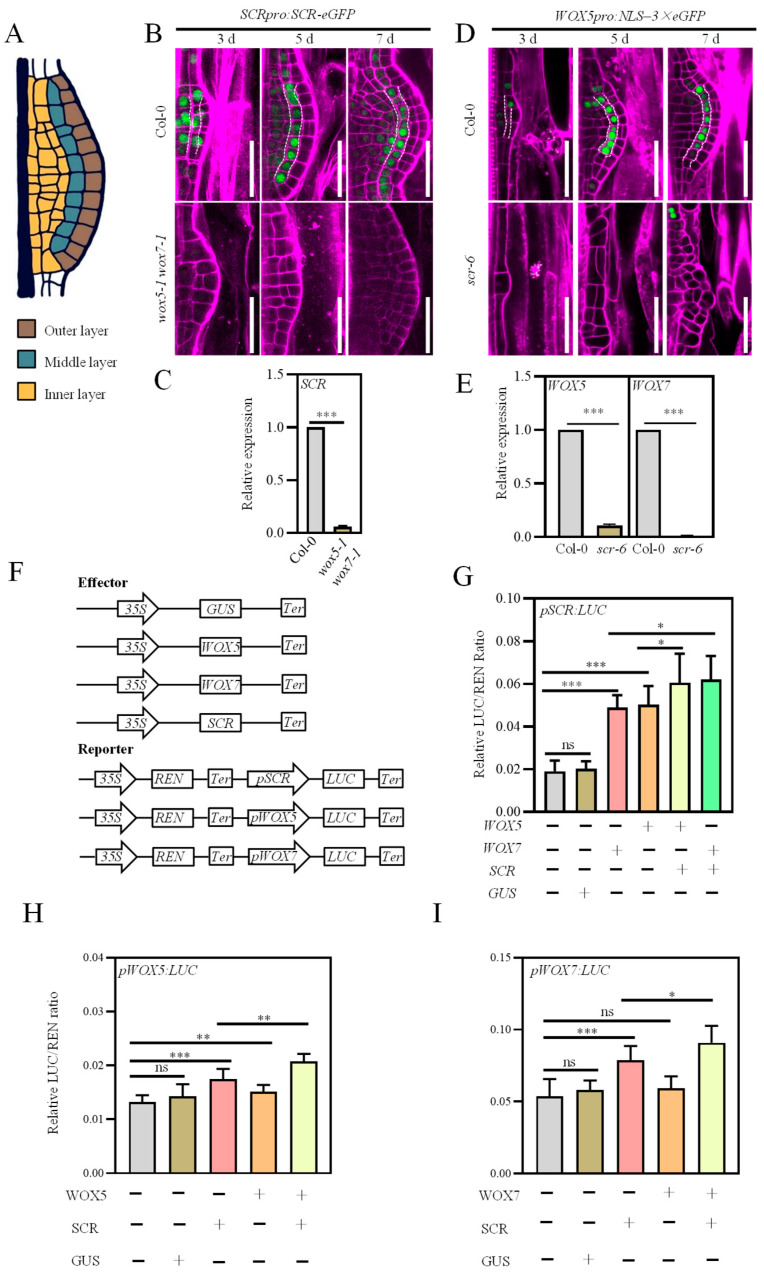
Mutual transcriptional regulation between WOX5/7 and SCR in callus formation. (**A**) Schematic representation of an early callus. Gray, blue and yellow areas denote the outer, middle and inner layers, respectively. (**B**) Representative confocal images of *SCRpro:SCR-eGFP* expression in 3- to 7-day-old calli from Col-0 and *wox5-1 wox7-1* mutants. Green, green florescent protein (GFP); Magenta, propidium iodide (PI). White dashed lines outline the middle cell layer. Scale bar, 50 µm. (**C**) RT-qPCR analysis of *SCR* expression in 5-day-old calli of Col-0 and *wox5-1 wox7-1* mutants. Data are mean ± SE (n = 4). *** *p* < 0.001 by two-sided Student’s *t*-test. (**D**) Representative confocal images of *WOX5pro:NLS–3×eGFP* expression in 3- to 7-day-old calli from Col-0 and *scr-6* mutants. White dashed lines outline the middle cell layer. Scale bar, 50 µm. (**E**) RT-qPCR analysis of *WOX5* and *WOX7* expression in 5-day-old calli of Col-0 and *scr-6* mutants. (**F**) Schematic of the effector and reporter constructs used for transient expression assays in (**G**–**I**). *35S*, constitutive cauliflower Mosaic virus (CaMV) *35S* promoter. Ter, *35S* terminator. (**G**–**I**) Transient dual-luciferase assays in *N. benthamiana* leaves co-transformed with the effector and reporter constructs as indicated. Results are presented as the ratio of LUC (firefly luciferase) to REN (renilla luciferase) activity. Data are mean ± SE (n = 3). * *p* < 0.05, ** *p* < 0.01, *** *p* < 0.001, ns (not significant) by two-sided Student’s *t*-test.

Single-cell transcriptome profiling has categorized callus into three developmental zones: the outer, middle, and inner cell layers (Figure 1A) [5]. Several transcription factors, including *WOX5/7* and *SCR*, have been shown to play essential roles in establishing pluripotency in the middle cell layer [15]. Consistent with previous findings, both *wox5-1 wox7-1* and *scr-6* mutants displayed delayed development and abnormal cell division patterns during callus formation compared with the wild type (WT) (Figure 1B,D) [15]. However, the *scr-6* calli exhibited much more severe defects in cell layering than those of *wox5-1 wox7-1* (Figure 1B,D). These observations suggest that *WOX5/7* and *SCR* play similar yet distinct roles in callus formation.

To investigate the molecular relationship between *WOX5/7* and *SCR*, we first examined the spatiotemporal expression of a translational reporter, *SCRpro:SCR-eGFP*, using confocal microscopy. In WT callus, SCR protein was initially distributed across all cell layers at 3 days after induction and became progressively restricted to the middle cell layer over time (Figure 1B). In contrast, SCR expression was barely detectable in the *wox5-1 wox7-1* double mutant (Figure 1B). The observed changes in *SCR* expression were further confirmed by Reverse Transcription Quantitative Real-Time PCR (RT-qPCR) (Figure 1C). Collectively, these results demonstrate that *WOX5/7* are required to maintain *SCR* expression in the callus.

We next asked whether *SCR*, in turn, regulates *WOX5/7* expression. As previously reported [5,23], the transcriptional reporter *WOX5pro:NLS-3×eGFP* exhibited an expression pattern similar to that of *SCRpro:SCR-eGFP*, with enrichment in the middle cell layer during callus development (Figure 1D). However, this expression was markedly reduced in the *scr-6* loss-of-function mutant compared to WT (Figure 1D). RT-qPCR analysis further confirmed the downregulation of both *WOX5* and *WOX7* in *scr-6* (Figure 1E), suggesting that *SCR* positively regulates *WOX5/7* expression. These findings are consistent with previous reports indicating that *SCR* activates *WOX5* expression during callus development [15].

To further validate the mutual transcriptional regulation between *WOX5/7* and *SCR*, we conducted transient dual-luciferase assays in *Nicotiana benthamiana* leaves. Transformation of *WOX5* or *WOX7* alone significantly activated *pSCR:LUC* expression, confirming that *WOX5/7* transcriptionally activate *SCR* (Figure 1F,G). Interestingly, co-transformation of *SCR* with *WOX5* or *WOX7* further enhanced *pSCR:LUC* activation beyond the level induced by *WOX5* or *WOX7* alone (Figure 1F,G), implying an additional regulatory layer beyond direct transcriptional control. Similarly, co-transformation of *SCR* with *WOX5* or *WOX7* resulted in stronger activation of *pWOX5:LUC* or *pWOX7:LUC* compared to transformation of *SCR* alone (Figure 1F,H,I). Together, these results support a model of reciprocal transcriptional reinforcement between *WOX5/7* and *SCR* during callus development.

### 3.2. Physical Interaction of WOX5/7 with SCR

**Figure 2:**
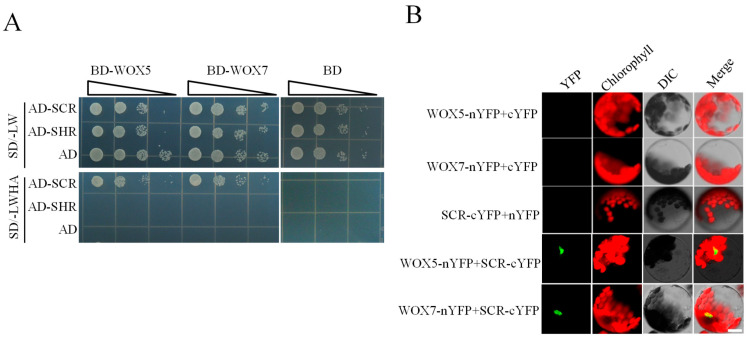
Physical interaction between WOX5/7 and SCR. (**A**) Y2H assay demonstrating the interaction between WOX5/7 and SCR. Yeast cells expressing the indicated fusion proteins were serially diluted and spotted onto synthetic defined (SD) medium lacking Leu and Trp (–LW) or SD medium lacking Leu, Trp, His, and Ade (–LWHA). AD, activation domain; BD, DNA-binding domain. (**B**) BiFC assay confirming the formation of the WOX5/7–SCR complex in *Arabidopsis* leaf protoplasts. Green, complemented YFP signal; Red, chlorophyll autofluorescence; DIC, differential interference contrast. Experiments were independently repeated three times with similar results. Scale bar: 10 μm.

The synergistic effect observed in the transient transcriptional assays prompted us to test for a potential protein–protein interaction between WOX5/7 and SCR. To experimentally test this hypothesis, we employed Yeast Two-Hybrid (Y2H) and Bimolecular Fluorescence Complementation (BiFC) assays. In the Y2H system, co-transformation of *WOX5* or *WOX7* with *SCR* supported robust yeast growth on stringent quadruple dropout medium (SD/-Ade/-His/-Leu/-Trp). In contrast, neither *WOX5* nor *WOX7* interacted with *SHORT ROOT* (*SHR*) under the same conditions (Figure 2A). This result indicates a specific protein–protein interaction between WOX5/7 and SCR. To validate this interaction in planta, we conducted BiFC assays in *Arabidopsis* leaf protoplasts. When SCR-cYFP was co-expressed with WOX5-nYFP or WOX7-nYFP, we observed reconstitution of YFP fluorescence specifically in the nucleus (Figure 2B). No signal was detected in the negative controls. Taken together, these results from two independent methods conclusively demonstrate that both WOX5 and WOX7 can directly interact with SCR in the nucleus.

### 3.3. WOX5/7 and SCR Co-Regulate a Substantial Number of Cell Cycle Related Genes

**Figure 3:**
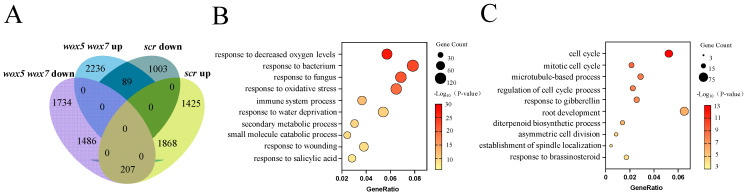
Comparative transcriptomic analysis of *scr* and *wox5 wox7* mutants. (**A**) Venn diagram illustrating the overlap between differentially expressed genes (DEGs) in *scr-6* and *wox5-1 wox7-1* mutants compared to Col-0 wild type (|log_2_FC| ≥ 1, adjusted *p* ≤ 0.05). Up, upregulated DEGs; down, downregulated DEGs. (**B**) Top 10 Gene Ontology (GO) terms for biological processes enriched among the commonly up-regulated DEGs in both mutants. (**C**) Top 10 GO terms for biological processes enriched among the commonly down-regulated DEGs in both mutants.

To elucidate the molecular mechanisms governed by the *WOX5/7-SCR* regulatory circuit, we conducted genome-wide transcriptome analyses of *wox5 wox7* and *scr* mutant callus. In the *wox5-1 wox7-1* mutant, 3427 genes were down-regulated and 4193 genes were up-regulated relative to the wild type, whereas the *scr-6* mutant exhibited 2578 down-regulated and 3500 up-regulated genes (|log_2_FC| ≥ 1, adjusted *p* ≤ 0.05). Among these, 1486 and 1868 differentially expressed genes (DEGs) were commonly down-regulated and up-regulated, respectively, in both mutants (Figure 3A and Table S3). Comparative transcriptomic analysis further revealed a statistically significant overlap of DEGs between *wox5-1 wox7-1* and *scr-6* (Fisher’s exact test, *p* < 0.05), indicating shared downstream regulatory pathways affected by both mutations.

Gene Ontology (GO) enrichment analysis showed that the majority of the top 10 enriched terms for the commonly up-regulated genes in *wox5-1 wox7-1* and *scr-6* mutants were associated with responses to biotic or abiotic stresses (Figure 3B). In line with the established function of *WOX5/7* in promoting callus cell division [23], commonly down-regulated genes were significantly enriched in functional categories related to the cell cycle and cell division (Figure 3C). Together, these transcriptomic data provide compelling evidence supporting the model that *WOX5/7* and *SCR* act in the same pathway to coordinately promote callus development.

### 3.4. The WOX5/7-SCR Module Drives Callus Cell Proliferation

**Figure 4:**
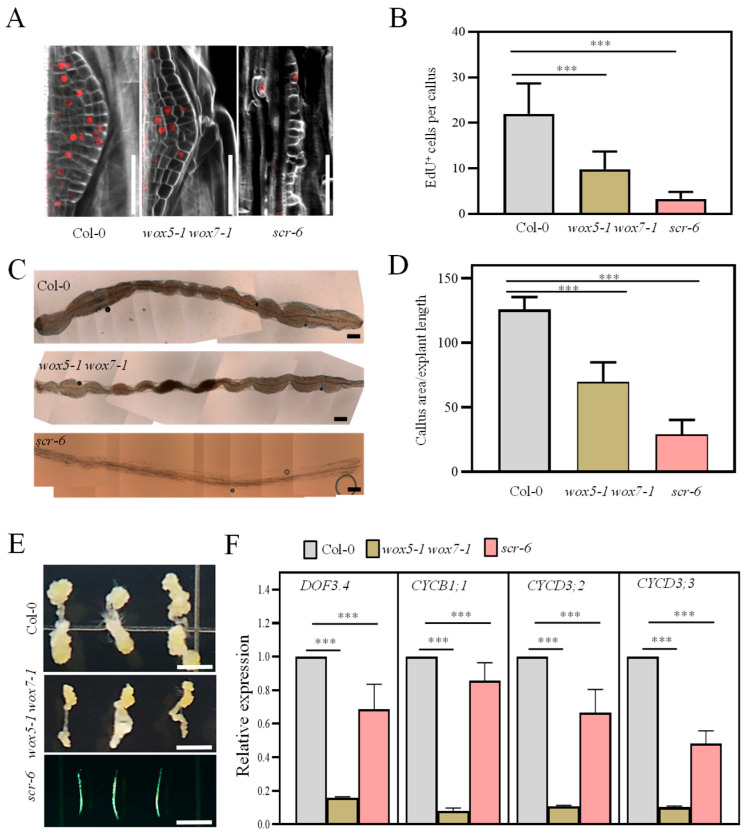
The *WOX5/7-SCR* Module promotes callus cell proliferation. (**A**) Representative confocal images of 7-day-old calli grown on CIM, stained with EdU to label proliferating cells. Note that due to its limited absorption efficiency, EdU labeling was primarily confined to the callus near the hypocotyl incision. (**B**) Quantification of EdU-positive cells from (**A**). Data are presented as mean ± SE (n = 15 calli per genotype). (**C**) Representative microscopy images showing the overall morphology of hypocotyl explants of the indicated genotypes cultured on CIM for 11 days. (**D**) Quantification of callus area from (**C**). Data are presented as mean ± SE (n > 15 explants per genotype). (**E**) Representative stereomicroscope images of calli from the indicated genotypes after three weeks of culture on CIM (n > 15 explants per genotype). Note the scarce callus formation on explant hypocotyls. (**F**) RT-qPCR analysis of the relative transcript levels of cell cycle-related genes *DOF3.4*, *CYCB1;1*, *CYCD3;2* and *CYCD3;3* in 5-day-old calli of the indicated genotypes. Data are presented as mean ± SE (n = 3 biological replicates). *** *p* < 0.001, ns (not significant) by two-sided Student’s *t*-test. Scale bars: 50 μm in (**A**), 600 μm in (**C**) and 0.5 cm in (**E**).

To investigate the biological significance of the co-regulation of cell cycle genes by *WOX5/7* and *SCR*, we analyzed cell cycle progression in their respective loss-of-function mutants. Calli from *wox5-1 wox7-1* and *scr-6* mutants were pulse-labeled with 5-ethynyl-2′-deoxyuridine (EdU) for five days on CIM. Since EdU is incorporated during DNA synthesis, this assay specifically identifies cells in the S-phase of the cell cycle. We observed a significant reduction in the number of EdU-positive cells in both mutants compared to the WT, indicating impaired S-phase progression (Figure 4A,B). Consistent with this cell cycle defect, both mutants also developed notably smaller calli (Figure 4C–E). In particular, *scr-6* produced almost no visible callus after three weeks of culture (Figure 4E).

We have previously shown that *WOX5/7* promote cell division by sustaining the expression of cell cycle-related genes, including *DOF3.4*, *CYCB1;1*, *CYCD3;2*, and *CYCD3;3* [24]. Here, RT-qPCR analysis revealed that the expression of all four genes was similarly downregulated in *scr-6* callus, mirroring the expression pattern in *wox5-1 wox7-1* (Figure 4F) [23]. Notably, *CYCD3;2* and *CYCD3;3* were also significantly downregulated in the transcriptome profiling of both *wox5-1 wox7-1* and *scr-6* mutants (Table S3).

Taken together, our data demonstrate that *SCR* functions jointly with *WOX5/7* to promote callus proliferation by activating a core set of cell cycle genes.

## 4. Discussion

**Figure 5:**
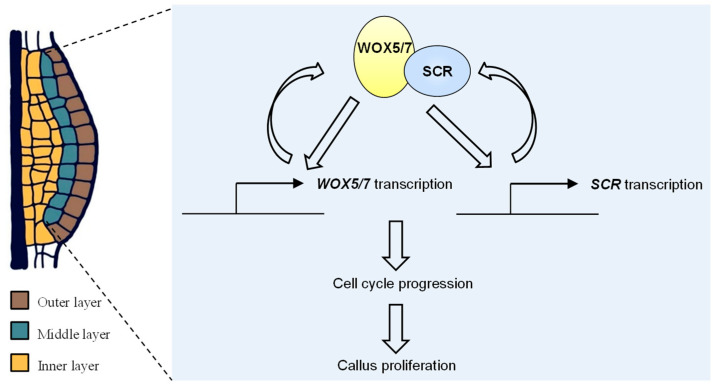
A working model for the coordination of *WOX5/7* and *SCR* in callus formation. The transcription factors WOX5/7 (yellow ellipse) and SCR (blue ellipse) form a transcriptional complex in the middle cell layer of callus. This complex cooperatively and efficiently activates its own expression, forming a positive feedback loop that drives callus proliferation.

The sustained proliferation of pluripotent cells is crucial for plant regeneration from callus, but its transcriptional control remains poorly understood. Here, we identify a core regulatory module wherein *WOX5/7* and *SCR* mutually activate each other’s expression. Furthermore, their proteins form a complex that reinforces this reciprocal transcription, establishing a positive feedback loop. This loop, in turn, drives callus proliferation by activating cell cycle-related genes (Figure 5).

### 4.1. A Positive Feedback Loop Stabilizes the Pluripotent Middle Layer

A key finding of our work is the reciprocal transcriptional activation between *WOX5/7* and *SCR*. While the regulation of *WOX5* by *SCR* has been previously suggested [15], our data provide direct genetic and molecular evidence for a fully reciprocal relationship. The loss of *SCR* abolishes *WOX5/7* expression (Figure 1D,E), and conversely, the *wox5 wox7* mutations lead to a significant reduction in *SCR* transcription (Figure 1B,C). This mutual dependency is further amplified at the protein level. The synergistic enhancement of each other’s promoters in transactivation assays (Figure 1F–I), coupled with the direct physical interaction between WOX5/7 and SCR proteins in the nucleus (Figure 2), strongly supports a model where these factors form a protein complex that stabilizes and reinforces their own transcription. This positive feedback loop likely serves as a robust molecular mechanism to maintain the middle callus layer, ensuring a stable source of pluripotent cells for subsequent organ regeneration.

### 4.2. The Potential of the WOX5/7-SCR Complex in Enhancing Crop Regeneration

In animals, a combination of four transcription factors (Oct4, Sox2, Klf4, and c-Myc) can reprogram differentiated somatic cells into pluripotent stem cells [25]. Among these, Oct4 and Sox2 form a heterodimer that serves as a cornerstone for the gene regulatory network governing pluripotency [26,27]. Analogous to this principle in animals, the overexpression of a fusion protein combining wheat GROWTH-REGULATING FACTOR 4 (GRF4) and its cofactor GRF-INTERACTING FACTOR 1 (GIF1) has been shown to substantially improve regeneration efficiency from callus in wheat [28]. These examples underscore the importance of enhancing protein complex activity to promote pluripotency acquisition and, consequently, regeneration efficiency.

In plants, research has demonstrated that the co-overexpression of *WOX5* and *SCR* significantly enhances shoot-regeneration efficiency in *Arabidopsis* [16]. Moreover, overexpression of *TaWOX5* alone significantly improves regeneration efficiency in wheat [29]. Therefore, by analogy to the successful GRF4-GIF1 fusion strategy and the documented synergy between *WOX5* and *SCR*, we propose that activating a WOX5/7-SCR chimera or co-expressing these genes could be a promising approach to enhance callus pluripotency and regeneration efficiency in crops.

## 5. Conclusions

Our study elucidates a core transcriptional module wherein a reciprocal *WOX5/7-SCR* feedback loop, reinforced at both the transcriptional and protein levels, drives callus proliferation by activating cell cycle genes. These findings significantly advance our understanding of the molecular mechanisms governing pluripotent cell growth during callus formation.

## Data Availability

The raw sequence data reported in this paper have been deposited in the Genome Sequence Archive (Genomics, Proteomics & Bioinformatics 2025) in National Genomics Data Center, China National Center for Bioinformation/Beijing Institute of Genomics, Chinese Academy of Sciences (GSA: CRA035560) that are publicly accessible at https://ngdc.cncb.ac.cn/gsa (accessed on 18 December 2025).

## Supplementary Materials

The supporting information can be downloaded at: https://www.mdpi.com/article/10.3390/plants15020210/s1.
